# Hydrophobic Tagging-Mediated Degradation of Transcription Coactivator SRC-1

**DOI:** 10.3390/ijms22126407

**Published:** 2021-06-15

**Authors:** So Ra Choi, Hee Myeong Wang, Min Hyeon Shin, Hyun-Suk Lim

**Affiliations:** 1 Department of Chemistry and Division of Advanced Material Science, Pohang University of Science and Technology (POSTECH), Pohang 37673, Korea; sora7116@postech.ac.kr (S.R.C.); hmwang@postech.ac.kr (H.M.W.); 2 POSTECH Biotech Center, Pohang 37673, Korea

**Keywords:** PROTACs, hydrophobic tagging, ubiquitin–proteasome system, ubiquitination, proteasomal degradation, cancer metastasis, SRC-1 transcriptional coactivator

## Abstract

Steroid receptor coactivator-1 (SRC-1) is a transcription coactivator playing a pivotal role in mediating a wide range of signaling pathways by interacting with related transcription factors and nuclear receptors. Aberrantly elevated SRC-1 activity is associated with cancer metastasis and progression, and therefore, suppression of SRC-1 is emerging as a promising therapeutic strategy. In this study, we developed a novel SRC-1 degrader for targeted degradation of cellular SRC-1. This molecule consists of a selective ligand for SRC-1 and a bulky hydrophobic group. Since the hydrophobic moiety on the protein surface could mimic a partially denatured hydrophobic region of a protein, SRC-1 could be recognized as an unfolded protein and experience the chaperone-mediated degradation in the cells through the ubiquitin–proteasome system (UPS). Our results demonstrate that a hydrophobic-tagged chimeric molecule is shown to significantly reduce cellular levels of SRC-1 and suppress cancer cell migration and invasion. Together, these results highlight that our SRC-1 degrader represents a novel class of therapeutic candidates for targeting cancer metastasis. Moreover, we believe that the hydrophobic tagging strategy would be widely applicable to develop peptide-based protein degraders with enhanced cellular activity.

## 1. Introduction

**Figure 1:**
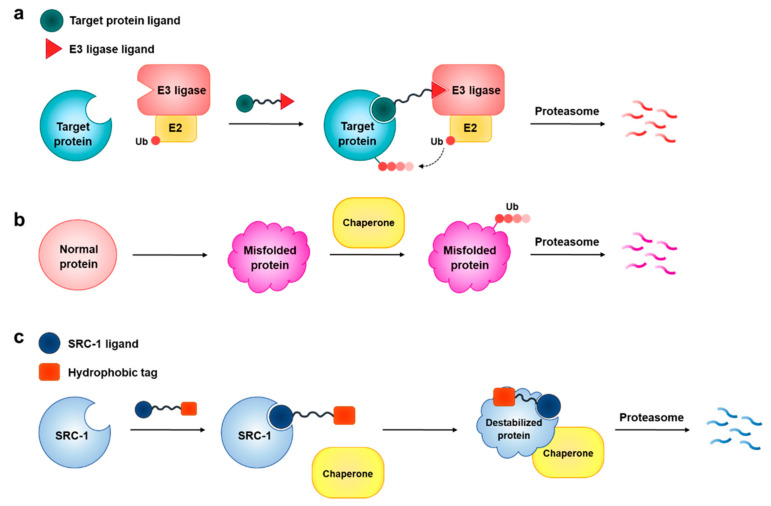
(**a**) Mechanism of PROTAC-mediated protein degradation. (**b**) Chaperone-assisted degradation of misfolded proteins through recognition of hydrophobic patches. (**c)** Schematic showing SRC-1 degradation with a hydrophobic tagging strategy.

Abnormally elevated levels of proteins are closely associated with various diseases [1,2,3]. Therefore, downregulation of such aberrantly activated proteins has emerged as a legitimate therapeutic strategy [4,5,6]. To this end, classical genetic techniques such as gene knockouts and small-interfering RNA (siRNA) have been widely used to suppress protein expression at the DNA or mRNA levels [7,8]. Although these conventional methods have been useful, their biological instability and difficulties in delivering such nucleic-acid-based therapeutics have hindered their clinical applications [9,10]. Alternatively, chemical knockdown methods that function at the post-translational level have been proposed as complementary strategies to circumvent these issues [11]. Proteolysis-targeting chimeras (PROTACs) are one such technology that induces the degradation of a target protein in the cells via the ubiquitin–proteasome system (UPS) [12,13,14]. PROTACs are heterobifunctional molecules composed of two recognition motifs, a ligand that binds to a target protein and a ligand that recruits E3 ubiquitin ligase (Figure 1a). Thus, PROTACs can bind to both the target protein and E3 ligase simultaneously and form a ternary complex, thereby leading to polyubiquitination of a target and subsequent elimination by the 26S proteasome. PROTACs have several important advantages compared with conventional biological methods. For example, PROTACs are not only cell permeable, but also relatively stable in biological systems. Moreover, they act in a catalytic manner in inducing protein degradation; PROTACs can exert sufficient pharmacological effects even at low dosages [5,14]. Despite these prominent benefits of PROTACs, their general applications might be limited. While there are over 600 E3 ligases in the human genome, only a few E3 ligases are targeted by current PROTACs, which are overexpressed in cancer cells (e.g., cereblon and Von Hippel–Lindau tumor suppressor). As a result, when target cells or tissues do not express such E3 ligases, it is challenging to develop PROTACs. Indeed, most PROTACs have been developed for cancer therapy [15,16,17,18].

In an effort to circumvent the restriction, we recently developed a new class of PROTACs based on the N-degron pathway [19]. The N-degron pathway is a ubiquitin-dependent proteolytic system for protein degradation through the recognition of N-terminal residues of proteins (called N-degrons) [20,21]. These N-degrons containing a few amino acids are recognized by ubiquitin ligase E3 component N-recognin (UBR) proteins, a unique class of E3 ligases that share the UBR box domain, for ubiquitination and proteasomal degradation. Therefore, the N-degron determines the half-life of substrate proteins. In contrast to the E3 ligases targeted by current PROTAC molecules, UBR proteins are ubiquitously expressed in most cells, and thus PROTACs based on the N-degron pathway could degrade proteins irrespective of cell type [22,23]. As such, this type of PROTACs could be generally applicable for targeting a wide range of diseases. In our previous study, we demonstrated the utility of PROTACs based on the N-degron pathway by developing a selective degrader (ND1-YL2) of steroid receptor coactivator-1 (SRC-1) [19]. SRC-1 is a transcription coactivator that interacts with various nuclear receptors (NRs) and transcriptional factors to regulate the transcriptional network [24,25]. It consists of an NR interaction domain, activation domain (AD) 3 in N-terminus, and AD1 and AD2 in C-terminus [26]. SRC-1 is a member of the p160 SRC family that includes its homologous proteins, such as SRC-2 and SRC-3 [25]. Abnormally elevated SRC-1 activity is found to be linked to cancer progression, recurrence, and poor survival rate [27,28,29]. Hence, downregulation of SRC-1 activity has emerged as a valid therapeutic strategy for the treatment of various cancers [30].

**Figure 2:**
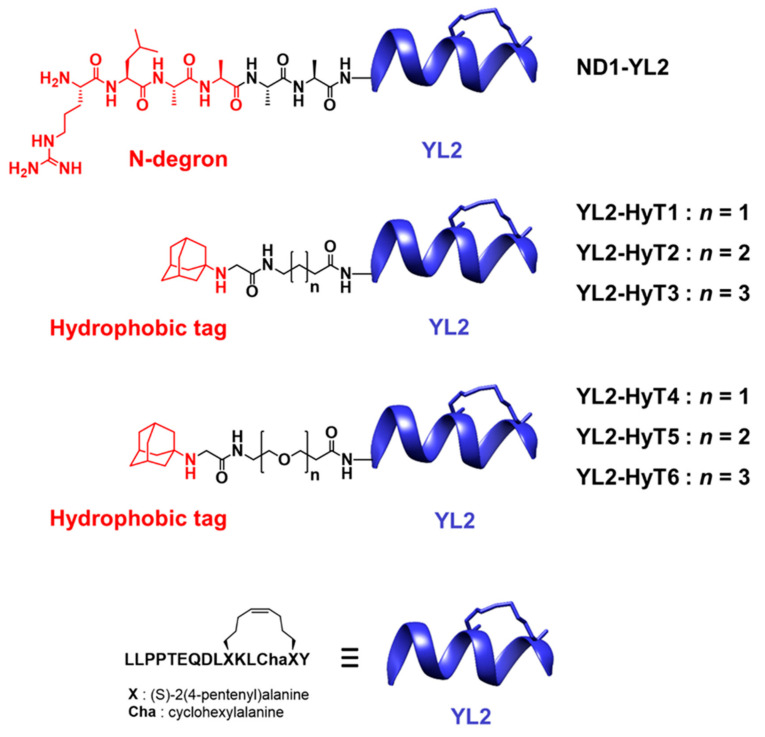
Chemical structures of ND1-YL2 and hydrophobic-tag-conjugated stapled peptides (YL2-HyT1–6).

The SRC-1 degrader ND1-YL2 is a chimeric molecule composed of a ligand (YL2) for SRC-1 [31] and a UBR E3 ligase binder [32] (Figure 2). YL2 is a cell-permeable, stapled peptide that binds selectively to SRC-1 with a *K_i_* value of 140 nM [19]. ND1-YL2 was generated by conjugating a tetrapeptide RLAA, the N-degron peptide as a binder for UBR. Thus, this bifunctional molecule enables the recruitment of SRC-1 to UBR protein, thereby promoting the formation of a ternary complex for polyubiquitination and proteasomal degradation of SRC-1. We found that ND1-YL2 effectively suppressed SRC-1-mediated transcriptional activity by inducing the degradation of cellular SRC-1, thereby resulting in inhibition of the invasion and migration of cancer cells [19]. Taken together, we successfully demonstrated that PROTACs based on the N-degron pathway could be a generally applicable strategy for targeted degradation of proteins regardless of cell types.

Although ND1-YL2 displays robust SRC-1 degradation and inhibitory activity on tumor metastasis, its further development as a therapeutic candidate is limited. Unfortunately, ND1-YL2 was found to be rapidly degraded within 10 min when administered in mice probably due to the metabolic instability of the N-degron peptide part. Note that the stapled peptide part (YL2) of ND1-YL2 was shown to have increased proteolytic resistance compared with the corresponding native peptide sequence [31]. In addition, ND1-YL2 exhibited significantly weaker cellular activity in degrading SRC-1 (DC_50_ ≈ 10 μM), compared with its in vitro binding activity (*K_i_* ≈ 320 nM) [19]. We reasoned that the discrepancy between in vitro binding activity and cellular activity might be due to its relatively low cell permeability. Since YL2 was shown to have reasonable cell penetration ability [31], the N-degron peptide moiety was thought to interfere with the cellular uptake of ND1-YL2. In this study, we explore a hydrophobic tagging strategy to develop SRC-1 degraders with improved cellular activity. Hydrophobic tagging is a chemical biology technology that enables the inducement of the degradation of a protein of interest in living cells. This system uses a bifunctional molecule that consists of a ligand for a target protein conjugated with a bulky hydrophobic tag (e.g., adamantyl group) [33,34,35,36,37]. If this chimeric molecule is added to cells, it binds to its target protein, and the hydrophobic tag acts like a degron. That is, the bulky hydrophobic moiety on the protein surface could mimic a partially denatured hydrophobic region of a protein. Note that exposure of internal hydrophobic regions of a protein is a hallmark of protein unfolding. These unfolded proteins are eliminated by the chaperone-mediated degradation by the UPS (Figure 1b) [38]. As such, upon binding of the bifunctional molecule to a protein of interest, the bulky hydrophobic moiety could be recognized as hydrophobic regions of misfolded or unfolded proteins in the cells, thereby resulting in the degradation of the protein. We envisaged that replacing the N-degron peptide part of **ND1-YL2** with a hydrophobic tag would considerably improve the cell permeability and metabolic stability (Figure 1c).

## 2. Results and Discussion

### 2.1. Design and Synthesis of Hydrophobic-Tagged SRC-1 Degraders

We sought to develop hydrophobic-tagged SRC-1 degraders by attaching the adamantyl group to YL2. We previously solved the high-resolution crystal structure of YL2 complexed with the PAS-B domain of SRC-1 showing that the N-terminal of stapled peptide is solvent exposed [31]. As such, the N-terminal position of YL2 was predicted to be suitable for conjugating a linker and the hydrophobic tag. Based on this structural information, we designed a series of adamantane-conjugated derivatives, YL2-HyT1–6 (Figure 2), with various linkers, such as aminobutanoic acid, aminovaleric acid, aminohexanoic acid, mono(ethylene glycol), di(ethylene glycol), and tri(ethylene glycol). For the preparation of YL2-HyT1–6, peptide sequences of YL2 were synthesized by standard fluorenylmethyloxycarbonyl (Fmoc) solid-phase peptide synthesis method. Two (S)-2-(4-pentenyl)alanine residues of peptide were cross-linked by ring-closing olefin metathesis reaction to form a stapled peptide. After coupling various kinds of linkers to the stapled peptide, the adamantyl group was introduced to a linker by amine substitution reaction to give hydrophobic-tagged stapled peptides ((Scheme S1 in the Supplementary Materials). These products were cleaved from the resin and purified by reverse-phase HPLC (Figure S1).

**Figure 3:**
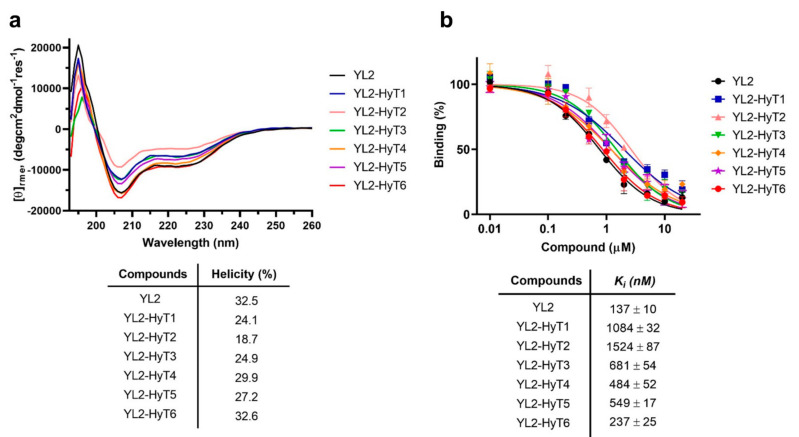
(**a**) CD spectra and calculated helicities (%) of YL2-HyT1–6 and YL2 (50 μM), (**b**) inhibition curves of YL2-HyT1–6 and YL2 for fluorescein-labeled STAT-6 peptide binding to SRC-1 determined by competitive FP assays. Error bars indicate standard deviation from three independent experiments.

Increased helical propensity in a stapled peptide is known to be an important factor for the improved binding ability of the stapled peptide for a target protein [31]. To examine whether the hydrophobic tagging affected the helical propensity of the stapled peptide YL2, circular dichroism (CD) spectroscopy was employed (Figure 3a). As expected, all the synthesized hydrophobic-tagged peptides displayed similar helical propensity, indicating that N-terminal modification with a linker and the adamantyl group had no effect on the helicity of the original stapled peptide YL2. Next, we conducted a competitive fluorescence polarization (FP) assay to measure the binding affinities of the synthesized compounds to SRC-1 (Figure 3b). Most compounds retained their abilities to bind to the recombinant PAS-B domain of SRC-1. This result was in good agreement with CD results. Among them, YL2-HyT6 with a tri(ethylene glycol) linker exhibited the best binding affinity (K_i_ = 237 nM), which was compatible with that of the original stapled peptide YL2 (K_i_ = 137 nM).

### 2.2. Hydrophobic-Tag-Coupled YL2 Molecules Degraded SRC-1

**Figure 4:**
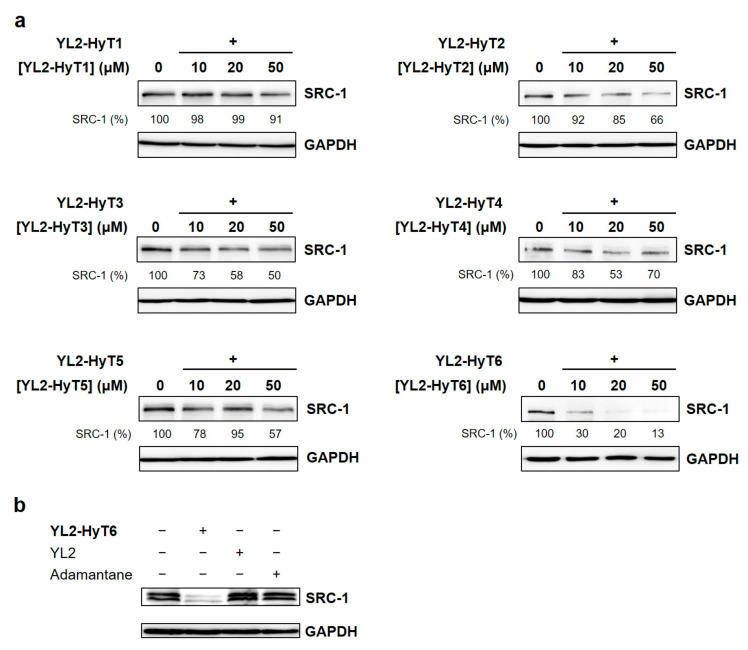
(**a**) Immunoblot analysis of SRC-1 levels in MDA-MB-231 cells after treatment with YL2-HyT1–6 for 18 h. SRC-1 levels (%) were normalized to GAPDH and DMSO controls. (**b**) Immunoblot analysis of SRC-1 after incubating MDA-MB-231 cells with YL2-HyT6 (20 μM), YL2 (20 μM), or adamantane (20 μM) for 18 h.

To investigate whether stapled peptides with a hydrophobic tag could degrade SRC-1 in cells, human triple-negative breast cancer (TNBC) MDA-MB-231 cells were treated with DMSO or various concentrations of the synthesized bifunctional compounds (YL2-HyT1–6). Cellular levels of SRC-1 were analyzed at various time points (12, 18, and 24 h) by immunoblotting. As depicted in Figure 4a (18 h), Figure S2 (12 h), and Figure S3 (24 h), some of the compounds induced SRC-1 degradation at micromolar concentrations. Consistent with the competitive FP assay results, YL2-HyT6 exhibited the most effective SRC-1 degradation activity at all three time points. As a control, we performed the same experiment with adamantane alone. As shown in Figure 4b, adamantane itself had no effect. Note that we previously found that the stapled peptide (YL2) lacking the hydrophobic tag was unable to induce SRC-1 degradation [19]. These results confirmed that the chimeric structure of YL2-HyT6 was essential for the hydrophobic-tagged strategy for targeted protein degradation. Notably, YL2-HyT6 reduced cellular SRC-1 levels with a DC_50_ value of ~5 μM (Figure S4), which is more than twice as good as that of ND1-YL2 (DC_50_ ≈ 10 μM) [19]. This result suggests that hydrophobic tagging leads to the improvement of cell permeability presumably due to the nonpeptidic character and hydrophobicity of the adamantyl group [36]. YL2-HyT6 was selected for further biological studies.

### 2.3. Evaluation of Cell Permeability and Serum Stability of YL2-HyT6

**Figure 5:**
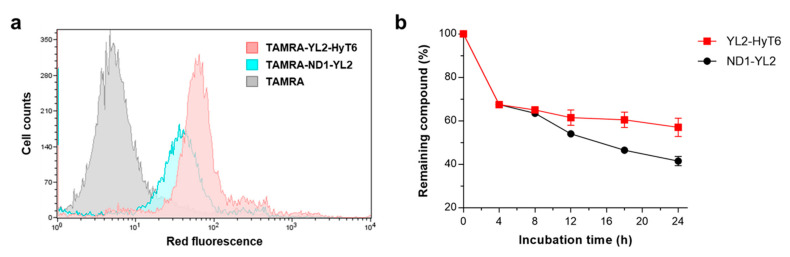
(**a**) Flow cytometry analysis of cellular uptake efficiency of TAMRA-YL2-HyT6 and TAMRA-ND1-YL2 in MDA-MB-231 cells. MDA-MB-231 cells were incubated with 10 μM compounds for 4 h at 37 °C. (**b**) Serum stabilities of YL2-HyT6 and ND1-YL2.

Since the increased protein degradation activity of YL2-HyT6 was thought to be due to its improved cell permeability, we sought to compare the cell permeability of YL2-HyT6 and ND1-YL2. To this end, we prepared carboxytetramethylrhodamine (TAMRA)-labeled derivatives of YL2-HyT6 and ND1-YL2 (Schemes S2 and S3). MDA-MB-231 cells were incubated with these fluorescently labeled compounds (TAMRA-YL2-HyT6 and TAMRA-ND1-YL2) and analyzed by flow cytometry. As shown in Figure 5a, the mean fluorescence intensity of the cells treated with TAMRA-YL2-HyT6 was higher than that of the cells with TAMRA-ND1-YL2, highlighting that the hydrophobic-tagged compound YL2-HyT6 indeed had better cell-penetrating ability than the peptide-based PROTAC ND1-YL2.

Next, we examined whether the hydrophobic-tagged compound YL2-HyT6 had enhanced stability to proteolytic degradation. YL2-HyT6 or ND1-YL2 was incubated in the media containing fetal bovine serum (FBS) for various time periods. Unsurprisingly, YL2-HyT6 showed improved stability relative to the peptide-based PROTAC (ND1-YL2), likely due to its nonpeptidic character (Figure 5b).

### 2.4. SRC-1 Degradation Relies on the Proteasome- and the Chaperone-Mediated Pathway

**Figure 6:**
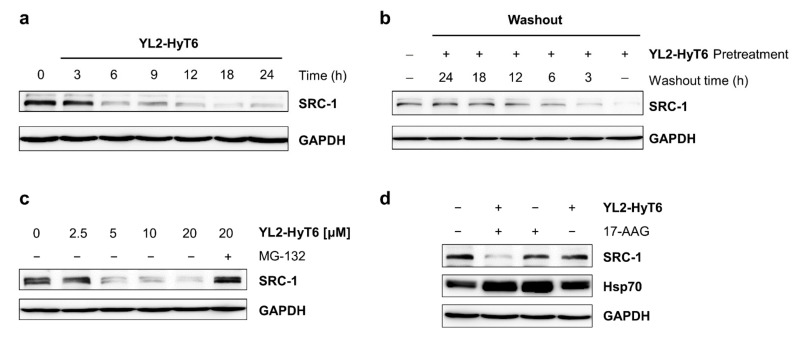
(**a**) Immunoblot analysis of SRC-1 levels in MDA-MB-231 cells after treatment with YL2-HyT6 (20 µM) for various time periods. (**b**) Immunoblot analysis of SRC-1 levels in MDA-MB-231 cells after washing out YL2-HyT6 (20 µM) at various time points. (**c**) Immunoblot analysis of SRC-1 after incubation of MDA-MB-231 cells with YL2-HyT6 or MG-132 (5 μM) for 18 h. (**d**) Immunoblot analysis of SRC-1 after incubating MDA-MB-231 cells with YL2-HyT6 (2.5 μM) or 17-AAG (1 μM) for 18 h.

To assess whether YL2-HyT6 degrades SRC-1 in a time-dependent fashion, MDA-MB-231 cells were treated with YL2-HyT6 (20 μM) for the indicated time period. Cellular levels of SRC-1 were then monitored by immunoblotting. As shown in Figure 6a, SRC-1 was evidently degraded after 6 h of YL2-HyT6 treatment. Detectable recovery of SRC-1 levels was not observed over 24 h. Next, we monitored whether SRC-1 was recovered after washing out YL2-HyT6. After treating the cells with YL2-HyT6 for 18 h, the compound was washed away. SRC-1 was shown to be recovered within 12 h (Figure 6b), indicating that SRC-1 degradation by YL2-HyT6 was reversible, unlike traditional genetic methods. To test whether YL2-HyT6 induced SRC-1 degradation by the proteasome-dependent pathway, MDA-MB-231 cells were cotreated with YL2-HyT6 and MG-132, a proteasome inhibitor. As expected, SRC-1 levels were not changed in the presence of MG-132, suggesting that YL2-HyT6 degrades SRC through the proteasome-mediated process (Figure 6c).

Protein degradation achieved by hijacking the unfolded protein pathway is known to be associated with molecular chaperones, such as heat shock proteins (HSPs). For example, Hsp70 recognizes exposed hydrophobic regions of unfolded or misfolded proteins and mediates polyubiquitination and proteasomal degradation. To examine this, cells were incubated with 17-(allylamino)-17-demethoxygeldanamycin (17-AAG), a well-known Hsp90 inhibitor that induces the upregulation of Hsp70 expression [39]. Immunoblot analysis showed that elevated levels of Hsp70 efficiently increased the cellular activity of YL2-HyT6, finally leading to SRC-1 degradation even at a concentration that did not affect SRC-1 levels previously (Figure 6d). These results were consistent with a previous study showing enhanced degradation activity toward the androgen receptor after cotreatment with an Hsp90 inhibitor [33]. These results indicate that SRC-1 degradation via hydrophobic tagging strategy can be induced through a chaperone-mediated ubiquitin proteasome system.

### 2.5. Evaluating the Effects of YL2-HyT6 on Cell Migration and Invasion In Vitro

**Figure 7:**
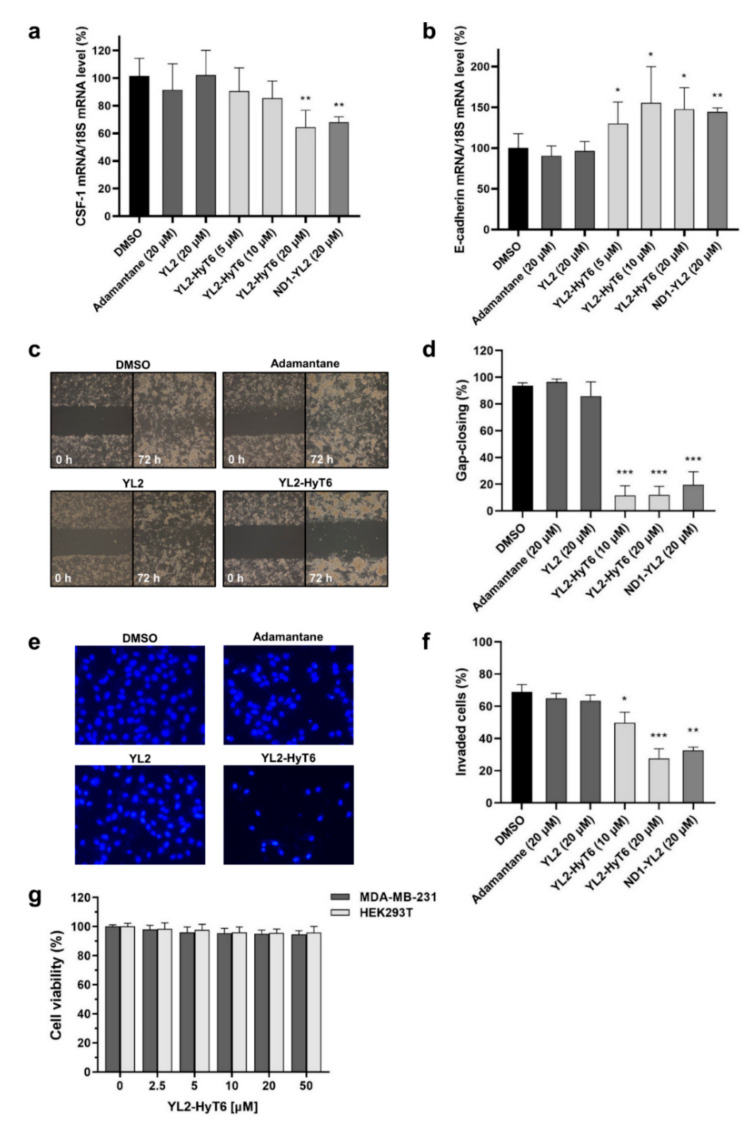
Cellular activities of YL2-HyT6. (**a**) *CSF-1* and (**b**) *E-cadherin* mRNA expression levels in MDA-MB-231 cells after treatment with DMSO, hydrophobic tag, YL2, ND1-YL2, or YL2-HyT6 for 18 h were analyzed by RT-qPCR. Results are presented as levels of *CSF-1* and *E-cadherin* genes in cells, which were normalized to the expression of the reference gene *18S*. (**c**) Representative images from wound healing assay showing changes in MDA-MB-231 cell migration after treatment with DMSO, hydrophobic tag (20 µM), YL2 (20 µM), or YL2-HyT6 (20 µM). Images of wound gap were taken at 0 and 72 h. (**d**) Quantitative analysis of wound gap closure in MDA-MB-231 cells. Results are presented as a percentage of gap-closed area. (**e**) Representative images of transwell invasion assay of MDA-MB-231 cells after treatment with DMSO, hydrophobic tag (20 µM), YL2 (20 µM), or YL2-HyT6 (20 µM) for 24 h. (**f**) Quantitation of the invasion assay in MDA-MB-231 cells. Results are expressed as a percentage of invaded cells. (**g**) The effect of YL2-HyT6 on the survival of MDA-MB-231 and HEK293T cells after treatment for 48 h. Error bars in data represent standard deviation from three independent experiments. Statistical comparisons were performed using a two-tailed Student’s *t*-test. *, *p* < 0.05; **, *p* < 0.01; and ***, *p* < 0.001 vs. DMSO control.

Next, we investigated the pharmacological effects of YL2-HyT6 on the SRC-1-mediated signaling. Overexpression of SRC-1 is frequently found in various cancers and linked to cell migration and invasion by regulating the expression of associated genes [27,28,40]. Depletion of SRC-1 is known to downregulate colony-stimulating factor-1 (*CSF-1*), which induces cell differentiation and migration [40]. In contrast, suppression of SRC-1 upregulates *E-cadherin*, a tumor suppressor gene and a key component of cell adhesion [27]. YL2-HyT6 would affect the SRC-1-dependent gene expression by downregulating SRC-1 in the cells. To examine this, TNBC MDA-MB-231 cells were treated with DMSO, a hydrophobic tag, YL2, ND1-YL2, or YL2-HyT6 for 18 h. *CSF-1* and *E-cadherin* messenger RNA (mRNA) levels were then evaluated by quantitative real-time polymerase chain reaction (RT-qPCR) and normalized to 18S levels. Consistent with previous studies [19,27,40], treatment of YL2-HyT6 resulted in a dose-dependent decrease in *CSF-1* (Figure 7a) and increase in *E-cadherin* (Figure 7b), whereas the hydrophobic tag and YL2 had no effect.

Finally, we tested whether YL2-HyT6 could suppress the migration and invasion of cancer cells by modulating SRC-1-mediated transcription. To this end, we performed a wound healing assay to measure cell migration in vitro. Invasive TNBC MDA-MB-231 cells were seeded in culture insert wells. After these cells formed a confluent monolayer, inserts were removed to make a scratch (an artificial gap). Subsequently, cells were treated with DMSO, a hydrophobic tag, YL2, ND1-YL2, or YL2-HyT6 for 72 h. The acquired cellular images were quantitatively analyzed to determine the cell migration. As shown in Figure 7c,d, YL2-HyT6 significantly inhibited gap closing. We then examined the effect of YL2-HyT6 on cancer cell invasion. MDA-MB-231 cells cultured in chambers containing Matrigel barrier were treated with DMSO, a hydrophobic tag (20 µM), YL2 (20 µM), ND1-YL2 (20 µM), or varying concentrations of YL2-HyT6. After incubating for 24 h, invading cells were quantified by immunofluorescent microscopy. Expectedly, YL2-HyT6 remarkably decreased the invasion of the invasive cancer cells (Figure 7e,f), which was consistent with the cell migration assay results (Figure 7c,d). It is noteworthy that the hydrophobic-tagged compound YL2-HyT6 displayed better cellular activities in suppressing cancer cell migration and invasion compared with peptide-based PROTAC (ND1-YL2) as anticipated (Figure 7c–f, Figures S6 and S7). Next, we investigated whether the suppression of cell invasion and migration by YL2-HyT6 resulted from its cytotoxicity. As shown in Figure 7g, reduction in SRC-1 levels had little effect on cell viability. This observation suggests that SRC-1 as a transcription coactivator plays a major role in promoting cancer metastasis, rather than in mediating cancer cell growth and proliferation, consistent with previous studies (e.g., SRC-1 knockout experiments) [19,28,30]. Taken together, our results highlight that SRC-1 degradation by the hydrophobic tagging method represents a promising therapeutic strategy for targeting cancer metastasis. 

## 3. Material and Methods

### 3.1. Reagents and General Methods

Rink amide MBHA resin (0.52 mmol/g) and Fmoc-protected amino acids were purchased from BeadTech (Gyeonggi, Korea). All other chemical reagents were obtained from commercial suppliers (Sigma-Aldrich, St Louis, MO, USA), TCI (Tokyo, Japan), Alfa Aesar, HarefreerMA, USA), and Ambeed (Arlington Heights, IL, USA)) and used without further purification. Antibodies were purchased from the following commercial suppliers: anti-SRC-1 (Santa Cruz Biotechnology, Dallas, TX, USA, sc-32789), anti-Hsp70 (Cell Signaling Technology, Danvers, MA, USA, 4872S), anti-GAPDH (Santa Cruz Biotechnology, sc-32233), anti-mouse IgG-horseradish peroxidase (HRP) (Cell Signaling Technology, 7076S), and anti-rabbit IgG-HRP (Cell Signaling Technology, 7074S). Synthesized compounds were characterized with an Agilent 1220 LC system Ontario, CA, USA) using a C18 reverse-phase HPLC column (Eclipse XDB, 3.5 µm, 4.6 mm × 150 mm). A gradient elution of 10% to 100% B in 7 min (maintain 100% B until 10 min) was used at a flow rate of 0.7 mL/min (solvent A: H_2_O with 0.01% trifluoroacetic acid (TFA); B: acetonitrile with 0.01% TFA). Crude peptides were purified by preparative HPLC (YL9100 GPC system) using a C18 reverse-phase HPLC column (Eclipse XDB, 5 µm, 21.2 mm × 150 mm) with a linear gradient of 10% to 100% B by changing the solvent composition over 60 min. Matrix-assisted laser desorption ionization–time-of-flight mass spectrometry (MALDI–TOF MS) was performed on an Autoflex Speed LRF (Bruker, Billerica, MA, USA) using 2,5-dihydroxybenzoic acid as a matrix.

### 3.2. Peptide Synthesis and Purification

#### 3.2.1. Synthesis of YL2-coupled adamantane with different linkers, YL2-HyT1–6 and ND1-YL2

Rink amide MBHA resins (200 mg, 104 μmol) were swollen in dimethylformamide (DMF) (2 mL) in a 6 mL fritted syringe for 2 h (h) at room temperature (rt). After removing the Fmoc protecting group with 20% piperidine in DMF (10 min × 2), Fmoc-protected amino acid (5 equiv) was coupled to the NH_2_ functional group on resins in the presence of 1-hydroxybenzotriazole hydrate (HOBt, 5 equiv); 2-(1*H*-benzotriazol-1-yl)-1,1,3,3-tetramethyluronium hexafluorophosphate (HBTU, 5 equiv); and *N,N*-diisopropylethylamine (DIPEA, 10 equiv) in DMF (2 mL) at rt. After shaking for 2 h, the reaction mixture was drained, and resins were washed with DMF (5×), methanol (MeOH) (3×), dichloromethane (DCM) (3×), MeOH (3×), and DMF (5×). This peptide coupling process was repeated until desired sequences (15 residues) of linear peptide were afforded. The peptide on resins was treated with 10 mM solution of Grubbs’ first-generation catalyst in anhydrous DCM for 2 h three times at rt. For the synthesis of YL2-HyT1–YL2-HyT6, after removing the Fmoc protecting group, resins were treated with 2 M bromoacetic acid (BAA, 20 equiv) and 2 M *N,N*-diisopropylcarbodiimide (DIC, 20 equiv) in DMF for 20 min at rt. Then 2 M adamantylamine (20 equiv) in DMF was coupled to resins for 4 h at 37 °C (Scheme S1). For the synthesis of ND1-YL2, two Fmoc-Ala-OHs were coupled to the N-terminal of the peptide as a linker. Then the N-degron peptide (RLAA) was synthesized with the same peptide coupling reaction. Next, products on resins were cleaved by treating with 2 mL of a cleavage cocktail (95% TFA, 2.5% H_2_O, and 2.5% triisopropylsilane (TIS)) for 2 h at rt. The crude product was purified by reverse-phase HPLC. The purity and identity were determined by LC and MALDI–TOF MS (Figure S1). 

#### 3.2.2. Synthesis of TAMRA-labeled compounds (TAMRA-YL2-HyT6 and TAMRA-ND1-YL2)

After swelling Rink amide MBHA resins (100 mg, 52 μmol) in DMF (1 mL) in a 6 mL fritted syringe for 2 h at rt, the Fmoc protection group was removed with 20% piperidine in DMF (10 min × 2). These resins were treated with N-alpha-Fmoc-N-epsilon-(4-methyltrityl)-L-lysine (Fmoc-Lys(Mtt)-OH, 5 equiv) in the presence of HOBt (5 equiv), HBTU (5 equiv), and DIPEA (10 equiv) in DMF (1 mL) at rt. After shaking for 2 h, the reaction mixture was drained, and resins were washed with DMF (5×), MeOH (3×), DCM (3×), MeOH (3×), and DMF (5×). After removing the Fmoc protecting group, 6-(Fmoc-amino)hexanoic acid (Fmoc-6-Ahx-OH) was coupled to the N-terminal of the peptide as a linker with the same peptide coupling reaction. After removing the Fmoc protecting group, the peptide was synthesized at the same peptide coupling conditions described in Section 2.1. The Mtt protection group was then deprotected with a solution of 2% TFA and 2% TIS in DCM at rt (2 min × 10). For neutralization, these resins were treated with 10% DIPEA in DMF (1 mL) for 1 h at rt. Then 5-(and-6)-carboxytetramethylrhodamine (5(6)-TAMRA) was coupled in the presence of 1-hydroxy-7-azabenzotriazole (HOAt) (2 equiv), 1-[bis(dimethylamino)methylene]-1H-1,2,3-triazolo[4,5-b]pyridinium 3-oxide hexafluorophosphate (HATU) (2 equiv), and DIPEA (4 equiv) in DMF (1 mL) for 3 h at rt with blocking light (Schemes S2 and S3). The product was cleaved from resins by treating with 1 mL of a cleavage cocktail solution. Subsequently, the crude product was purified by reverse-phase HPLC, and its purity and identity were determined by LC and MALDI–TOF (Figure S5).

### 3.3. Circular Dichroism (CD) Measurement

Lyophilized compounds (YL2 and YL2-HyT1 through YL2-HyT6) were dissolved in 30% PBS (pH 7.4) and 70% acetonitrile solution to a final concentration of 50 μM. CD spectra were measured with a Jasco J-815 CD spectropolarimeter using a quartz cuvette (2 mm path length). These spectra were averages of five successive accumulations with a scan rate of 100 nm/min. Raw data were converted in terms of per-residue molar ellipticity (deg·cm^2^·dmol^−1^·residue^−1^) as calculated per mole of amide groups present and normalized by the molar concentration of peptides. Smoothing and correction of background spectra were performed with Origin Pro 8.0 (OriginLab Corporation, Northampton, MA, USA). Then α-helical propensities of YL2 and YL2-HyT1 through YL2-HyT6 were calculated as previously reported [41].

### 3.4. Protein Expression and Purification

A plasmid expressing the PAS-B domain of human SRC-1 (residues 257–385) tagged with His6 was provided by John A. Robinson (University of Zürich at Switzerland). It was transformed into Rosetta *E. coli* cells. SRC-1 protein purification was performed as described previously [42].

### 3.5. Competitive Fluorescence Polarization

After incubating 100 nM of fluorescein-labeled 15-mer STAT-6 peptide with 1 μM of PAS-B domain of SRC-1 in a binding buffer (50 mM HEPES pH 7.4, 150 mM NaCl, 3.4 mM EDTA, and 0.01% Tween 20) in a black Greiner 384-well plate for 30 min, indicated concentrations of YL2 and YL2-HyT1 through YL2-HyT6 were added to each well. After incubation for another 1.5 h, fluorescence polarization was measured on a Tecan Infinite F200 Pro microplate reader (excitation wavelength: 485 nm, emission wavelength: 535 nm). The *K*_i_ values of the compounds in the competition assay were determined using the following equation [43].

(1) $$K_{i}=\frac{{\left[I\right]}_{50}}{\frac{{\left[L\right]}_{50}}{K_{D}}+\frac{{\left[P\right]}_{0}}{K_{D}}+1}$$

where [I]_50_ is the concentration of the free inhibitor at 50% inhibition, K_D_ is the dissociation constant of the protein–ligand complex, [L]_50_ is the concentration of the free labeled ligand at 50% inhibition, and [P]_0_ is the concentration of the free protein at 0% inhibition.

### 3.6. Cell Culture

MDA-MB-231 and HEK293T cells were cultured in a medium containing Dulbecco’s modified eagle medium (DMEM) supplemented with 1% penicillin–streptomycin (PS) and 10% FBS with 5% CO_2_ at 37 °C.

### 3.7. Immunoblotting

MDA-MB-231 cells were seeded into 6-well pates (Corning, 3506) at a density of 6 × 10^5^ cell per well. After incubation at 37 °C for 24 h, cells were treated with synthesized compounds in Opti-MEM medium for 12, 18, or 24 h. Cells were washed twice with cold Dulbecco’s phosphate-buffered saline (DPBS) prior to cell lysis using a cell lysis buffer (1% Triton X-100, 150 mM NaCl, 50 mM Tris-HCl pH 7.6, 0.1% SDS, 0.5% sodium deoxycholate, and 1 × protease inhibitor cocktail) on ice. Cell lysates were centrifuged at 13,000 rpm at 4 °C for 15 min. The supernatant was collected, and protein concentration was determined using Pierce^TM^ 660nm protein assay. A 6 × SDS loading buffer was added to the cell lysate and incubated at 95 °C for 5 min. The same amount of protein was subjected to SDS-PAGE and transferred to PVDF membranes. After blocking membranes with 5% skim milk in TBST (Tris-buffered saline with 0.05% Tween 20), membranes were incubated with a primary antibody at 4 °C overnight. Membranes were then incubated with HRP-linked secondary antibody at rt for 1 h and developed with Pico ECL solution. In MG-132 and 17-AAG treatment experiments, MDA-MB-231 cells were cotreated with synthesized compounds and MG-132 (5 μM) or 17-AAG (1 μM) at 37 °C for 18 h.

### 3.8. Flow Cytometry

MDA-MB-231 cells (1 × 10^5^ cells/well) were seeded into a 24-well plate (Corning, 3527) and incubated at 37 °C for 24 h. Cells were then incubated with 10 μM of TAMRA dye-labeled compound solutions in Opti-MEM. After 4 h of incubation, cells were washed twice with DPBS buffer and collected by trypsinization. Resulting cells were again washed with cold DPBS twice and placed on ice. These cells were treated with propidium iodide (PI) (0.5 μg/mL) and subsequently analyzed with an LSRFortessa flow cytometer (BD Biosciences). PI-stained cells were excluded from the results.

### 3.9. Serum Stability

YL2-HyT6 and ND1-YL2 solutions (200 μL, 40 μM in DPBS containing 25% FBS) were incubated at 37 °C for different time periods (0–24 h). For the precipitation of serum proteins, each sample was treated with 100 μL of 15% TFA and incubated at 4 °C overnight. Final samples were then centrifuged at 15,000 rpm for 10 min and filtrated for analysis using LC–MS with a C18 column. The ratio of the remaining compound (%) was calculated by integrating the chromatographic peak of a compound and normalized in comparison with a control sample (incubated without FBS).

### 3.10. Quantitative Real-Time Polymerase Chain Reaction (RT-qPCR)

Quantitative real-time PCR was performed for *CSF-1*, *E-cadherin*, and *18S* (as a reference gene) mRNAs. MDA-MB-231 cells (1.2 × 10^5^ cells/well) were seeded into a 12-well plate and incubated at 37 °C with 5% CO_2_ for 24 h. These cells were treated with indicated compounds (YL2, adamantane, ND1-YL2, or YL2-HyT6) for 18 h in Opti-MEM medium. After cells were washed with cold DPBS, mRNAs were isolated from these cells using an AccuPrep Universal RNA extraction kit (Bioneer, Daejeon, Korea). Then 1.5 µg of mRNAs were reverse-transcribed using AccuPower RocketScript Cycle RT PreMix (Bioneer) to generate complementary DNAs. RT-qPCR for *CSF-1*, *E-cadherin*, and *18S* was performed with a StepOnePlus Real-Time PCR System (Applied Biosystems, Waltham, MA, USA) and SYBR Green mix (Applied Biosystems) according to the manufacturer’s instructions with gene-specific primers (Table 1).

### 3.11. Gap Closure Migration Assay

MDA-MB-231 cells (2.8 × 10^4^ cells/well) were seeded into Culture-Insert 2 wells (ibidi, 80209) placed in a 35 mm culture dish. After incubation at 37 °C with 5% CO_2_ for 24 h, Culture-Insert 2 wells were carefully removed from the culture dish with a sterilized tweezer. These cells were then treated with indicated compounds (YL2, adamantane, ND1-YL2, or YL2-HyT6) in Opti-MEM medium for 72 h. Cell images were obtained with a culture microscope (Olympus, Tokyo, Japan, CKX41). The cell-free area of the image was quantified with ImageJ software (NIH, Bethesda, MD, USA) [44]. The relative gap-closure area was calculated using the following equation: where A was the cell-free area.

(2) $$\mathrm{Gap}-\mathrm{Closure}\;\%=\frac{A_{t=0\;h}-A_{t=72\;h}}{A_{t=0\;h}}\times 100$$

### 3.12. Invasion Assay

Matrigel invasion chambers (Corning 354480) were placed in 24-well plates and rehydrated with FBS-free DMEM medium at 37 °C in a CO_2_ incubator for 2 h. After removing the medium, 4 × 10^4^ cells of MDA-MB-231 were seeded into Matrigel invasion chambers and control chambers (Corning, 354578) with 125 µL of Opti-MEM medium. Then another 125 µL of each compound (YL2, adamantane, ND1-YL2, or YL2-HyT6) in Opti-MEM medium was added to each chamber. The bottom of the well was filled with 500 µL of DMEM medium containing 10% FBS 1% PS. The chambers were then incubated at 37 °C with 5% CO_2_ for 24 h. After removing the medium, the chambers were washed with cold PBS. Then noninvading cells on the upper surface of the membrane were removed by scrubbing with a cotton swab. Cells on the bottom side of the membrane were fixed with 500 µL of 4% formaldehyde for 10 min at rt. After washing with PBS, the bottom side of the chamber was stained with 500 µL of Hoechst 33342 (5 µg/mL) for 5 min at 37 °C in the CO_2_ incubator. After washing with PBS, cells were observed with a fluorescence microscope (Nikon, Tokyo, Japan, Eclipse TE 2000). The percent invasion (% invasion) of each sample was determined with the following equation:

(3) $$\%\;\mathrm{Invasion}=\frac{Mean\;\#\;of\;cells\;invading\;through\;Matrigel\;chamber\;membrane}{Mean\;\#\;of\;cells\;migrating\;through\;control\;chamber\;membrane}\times 100$$

### 3.13. Cell Viability Assay

MDA-MB-231 (1 × 10^4^ cells/well) and HEK293T (1 × 10^4^ cells/well) cells were seeded into a 96-well plate (Corning, 3595) and incubated at 37 °C with 5% CO_2_ for 24 h. These cells were then washed twice with DPBS and treated with 1% DMSO or various concentrations of YL2-HyT6 for 48 h in Opti-MEM medium. The remaining cells were then used to measure cell viability using a Cyto X cell viability assay kit (LPS solution, Daejeon, Korea, CYT3000) following the manufacturer’s instruction.

## 4. Conclusions

In this work, we developed a hydrophobic-tagged bifunctional molecule (YL2-HyT6) as a selective SRC-1 degrader. YL2-HyT6 was found to have significantly increased cell permeability and proteolytic stability. As anticipated, this hydrophobic-tagged chimeric molecule indeed exhibited enhanced cellular activity in degrading SRC-1, in comparison with the previously reported peptide-based SRC-1 degrader. Therefore, YL2-HyT6 represents a novel class of SRC-1 degrader that enables the elimination of cellular SRC-1 by hijacking the unfolded protein degradation pathway. We also demonstrated that YL2-HyT6 efficiently suppressed cancer cell migration and invasion. Together, these results indicate that the chemical knockdown of cellular SRC-1 by the hydrophobic tagging method would be a viable therapeutic strategy for targeting cancer metastasis. Our study suggests that hydrophobic tagging could be a compelling strategy to develop peptide-based PROTACs with improved cell permeability and proteolytic stability.

## Figures and Tables

**Table 1:** List of primers used in real-time qPCR.

Gene	Forward primer (5′-3′)	Reverse primer (5′-3′)
*18S*	GAGGCCGTAGGCTTATTGTG	GAGTAGCTCATATGTCTTCCCTACCT
*CSF-1*	GTTTGTAGACCAGGAACAGTTGAA	CGCATGGTGTCCTCCATTAT
*E-cadherin*	TGCTGCAGGTCTCCTCTTGG	AGTCCCAGGCGTAGACCAAG

## Data Availability

Data are contained within the article or Supplementary Materials.

## Supplementary Materials

The following are available online https://www.mdpi.com/article/10.3390/ijms22126407/s1.
