# Supplementary material for: Hydrophobic Tagging-Mediated Degradation of Transcription Coactivator SRC-1
Source: Int J Mol Sci. 2021 Jun 15;22(12):6407. doi: 10.3390/ijms22126407 (PMC8232704; doi:10.3390/ijms22126407)

International Journal of *Molecular Sciences*

Supporting Information

Hydrophobic Tagging-Mediated Degradation of Transcription Coactivator SRC-1

So Ra Choi [1,†], Hee Myeong Wang [1,†], Min Hyeon Shin [1,2,*] and Hyun-Suk Lim [1,*]

[1] Department of Chemistry and Division of Advanced Material Science, Pohang University of Science and Technology (POSTECH), Pohang 37673, Korea; sora7116@postech.ac.kr (S.R.C.); hmwang@postech.ac.kr (H.M.W.)
[2] POSTECH Biotech Center, Pohang 37673, Korea
* Correspondence: minh0812@postech.ac.kr (M.H.S.); hslim@postech.ac.kr (H.-S.L.)
† These authors contributed equally to this work.

Citation: Choi, S.R.; Wang, H.M.; Shin, M.H.; Lim, H.-S. Hydrophobic Tagging-Mediated Degradation of Transcription Coactivator SRC-1. *Int. J. Mol. Sci.* **2021**, *22*, 6407. https://doi.org/10.3390/ijms22126407

Received: 22 May 2021
Accepted: 12 June 2021
Published: 15 June 2021

Publisher's Note: MDPI stays neutral with regard to jurisdictional claims in published maps and institutional affiliations.

Copyright: © 2021 by the authors. Licensee MDPI, Basel, Switzerland. This article is an open access article distributed under the terms and conditions of the Creative Commons Attribution (CC BY) license (http://creativecommons.org/licenses/by/4.0/).

Table of Contents

1. Synthesis of YL2-HyT1 – 6. (Scheme S1)……………..…………….……………………S2

2. MALDI-TOF MS and LC spectra of purified YL2-HyT1 – 6. (Figure S1)…………….S3

3. Western blot analysis of SRC-1 levels after treatment of YL2-HyT1 – 6 for 12 h. (Figure S2)…………………………………………………………………………………….S9

4. Western blot analysis of SRC-1 levels after treatment of YL2-HyT1 – 6 for 24 h. (Figure S3)……………………………………………………...……………….…...…S10

5. Quantitation of SRC-1 levels after treatment of YL2-HyT6 for 18 h. (figure S4)..…....S11

6. Synthesis of a TAMRA-labeled YL2-HyT6. (Scheme S2)……...................................S12

7. Synthesis of a TAMRA-labeled ND1-YL2. (Scheme S3)……………………….…....S13

8. MALDI-TOF MS and LC spectra of purified TAMRA-YL2-HyT6 and TAMRA-ND1-YL2. (Figure S5)……………………………………………………………...…..S14

9. Image from wound healing assay after treatment with ND1-YL2. (Figure S6)…...……S16

10. Image from transwell invasion assay after treatment with ND1-YL2. (Figure S7)……S17

Scheme S1. Synthesis of YL2-HyT1 – 6.

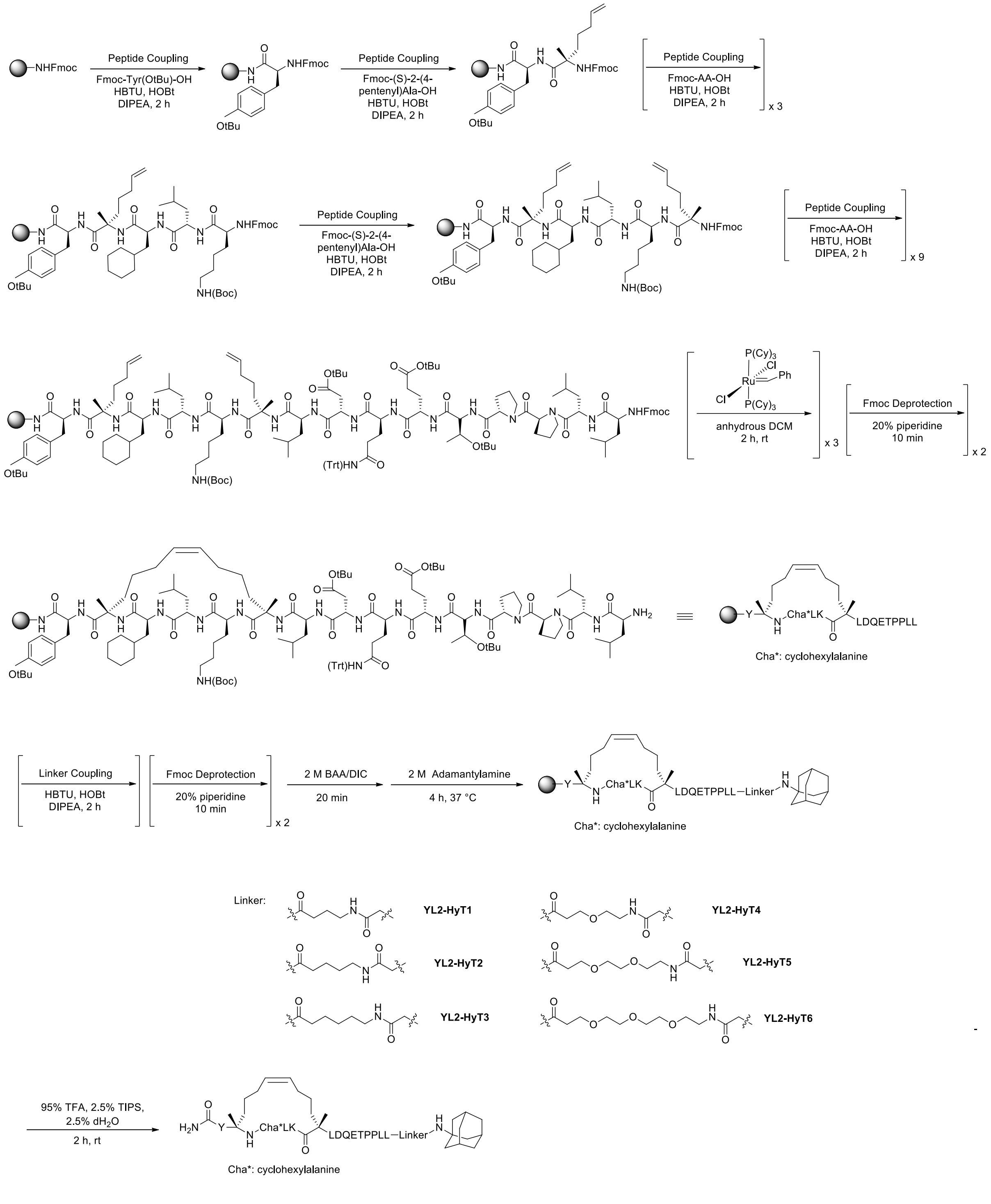

Figure S1. MALDI-TOF MS and LC data for purified YL2-HyT1 – 6.

YL2-HyT1

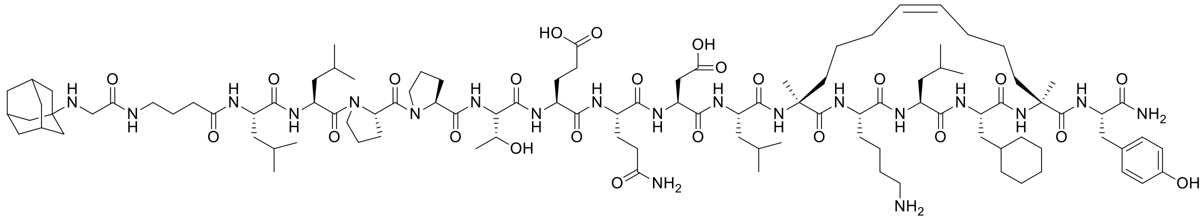

Calculated mass: 2107.27

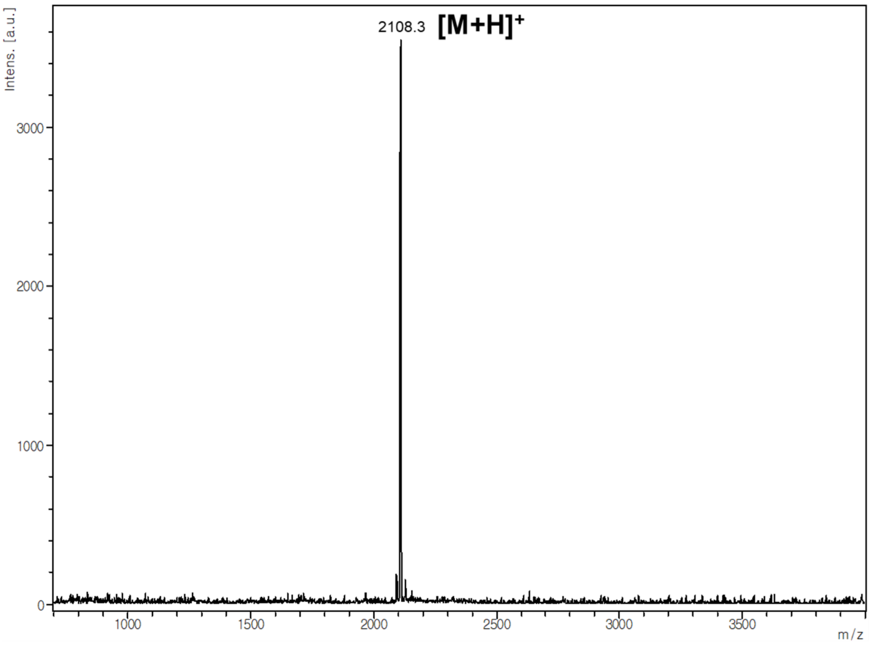

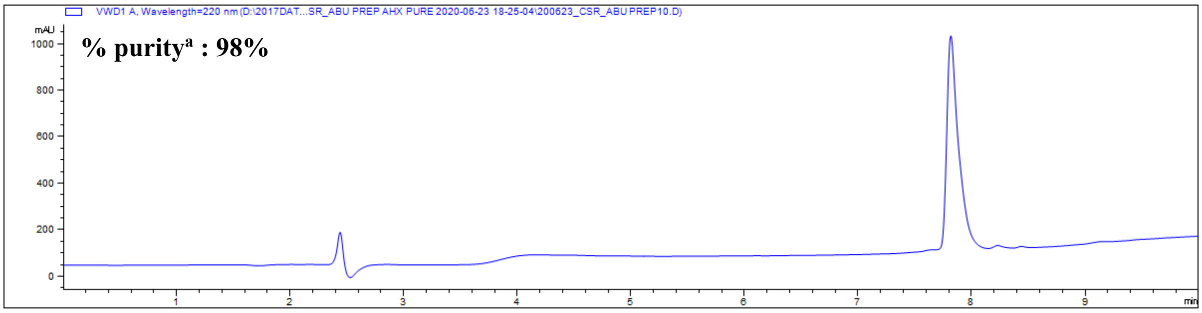

[a] Determined by analytical reversed-phase HPLC of a product

Figure S1. (Cont'd)

YL2-HyT2

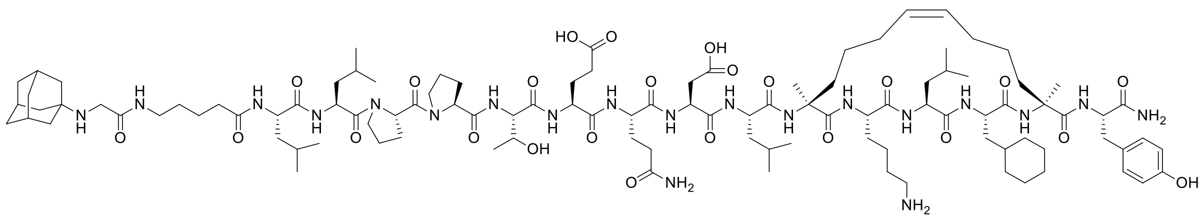

Calculated mass: 2121.29

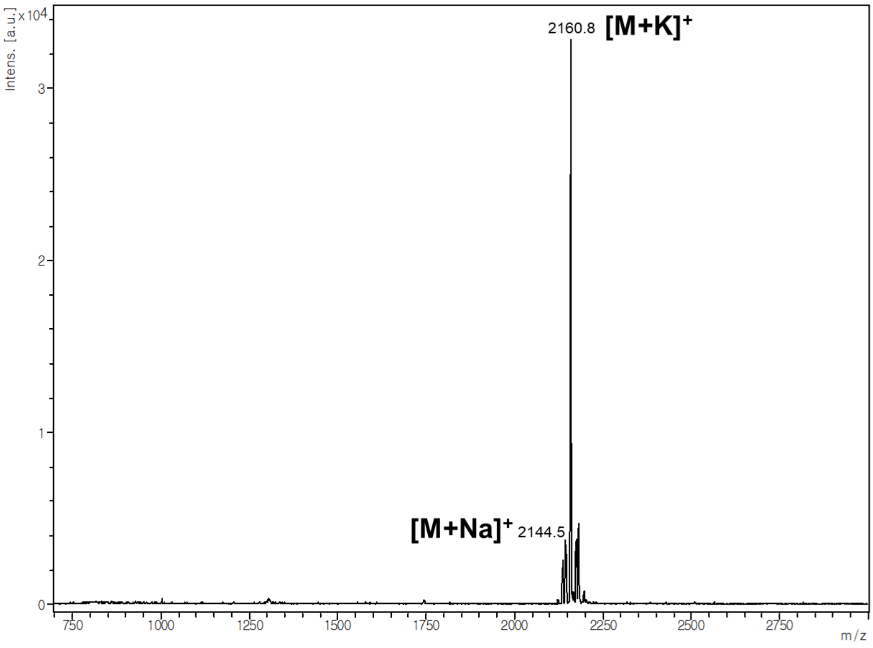

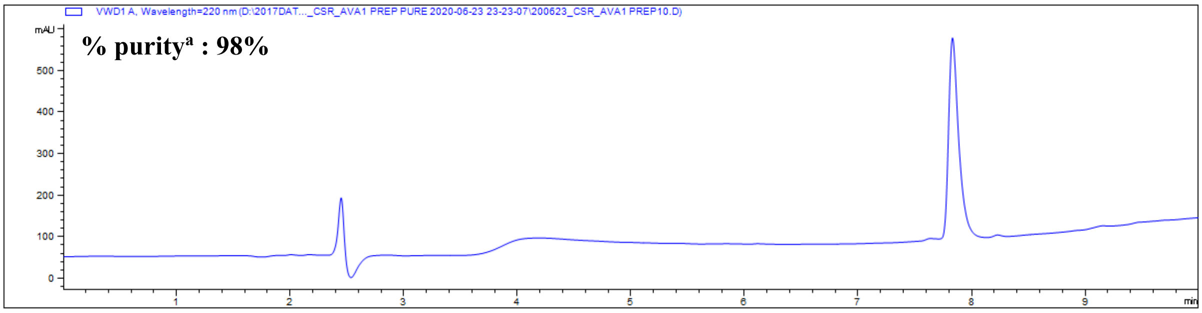

[a] Determined by analytical reversed-phase HPLC of a product

Figure S1. (Cont'd)

YL2-HyT3

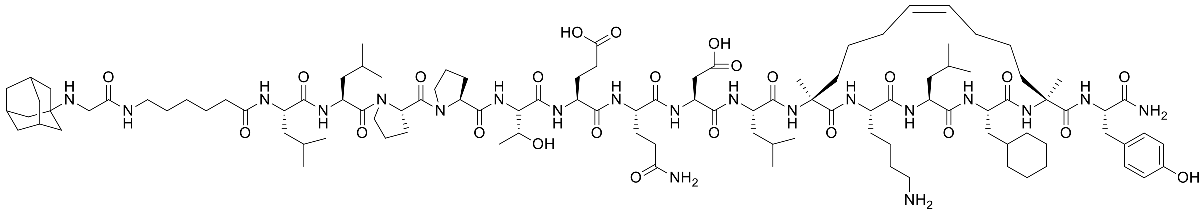

Calculated mass: 2135.30

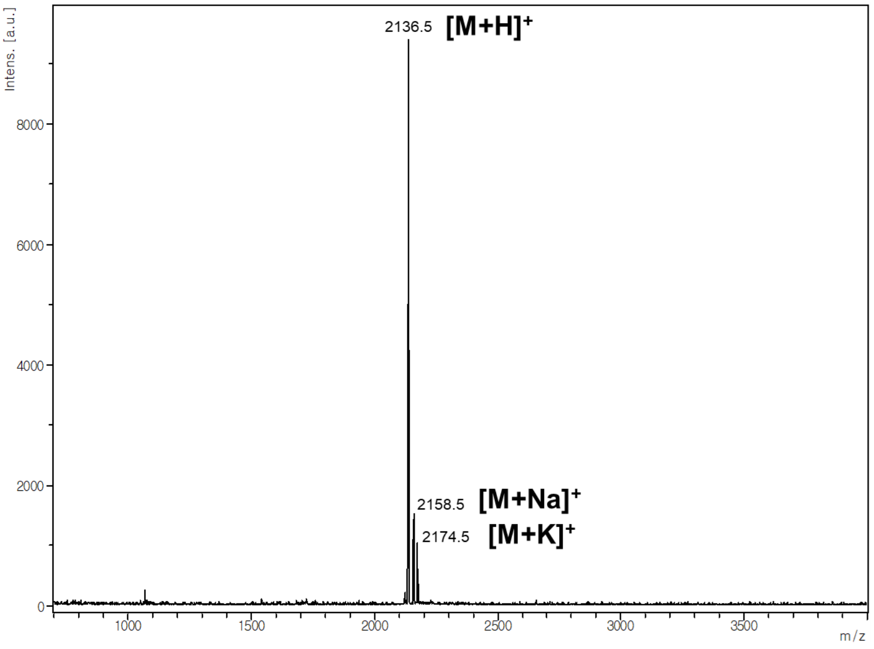

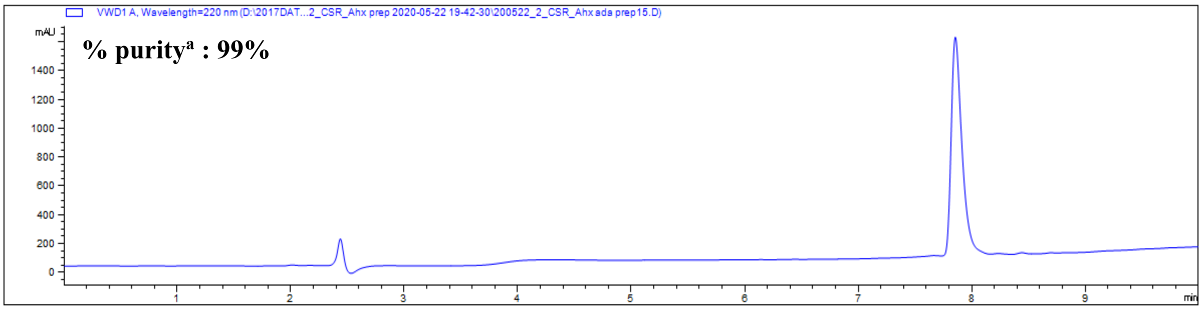

[a] Determined by analytical reversed-phase HPLC of a product

Figure S1. (Cont'd)

YL2-HyT4

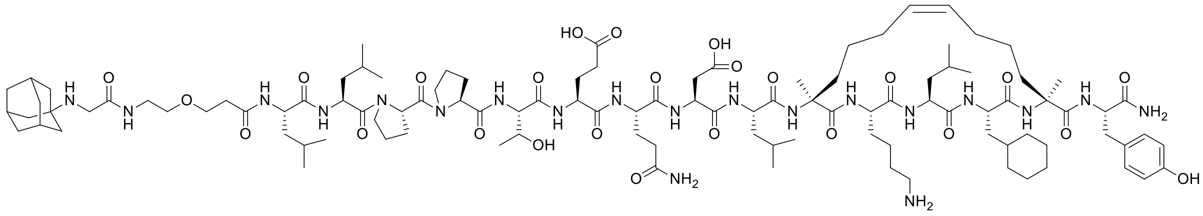

Calculated mass: 2137.28

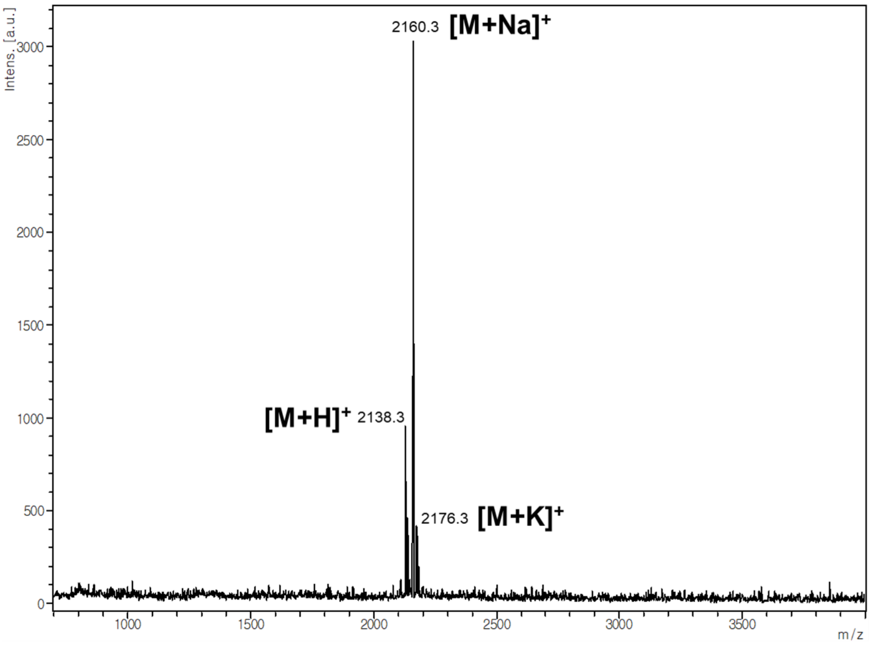

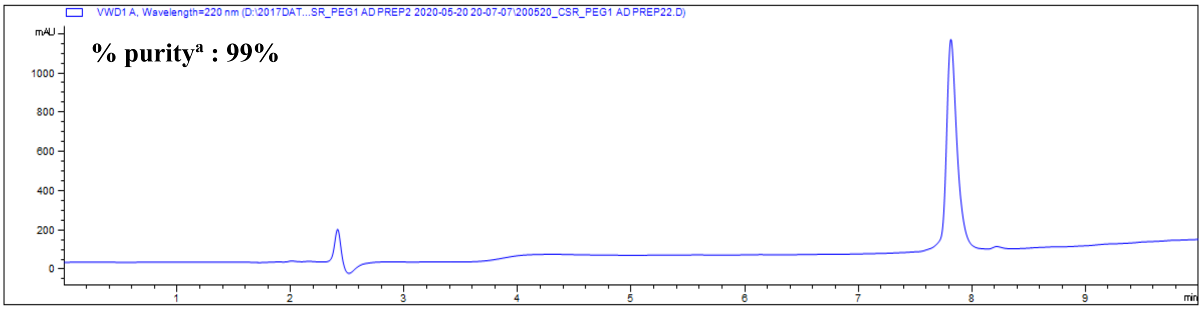

[a] Determined by analytical reversed-phase HPLC of a product

Figure S1. (Cont'd)

YL2-HyT5

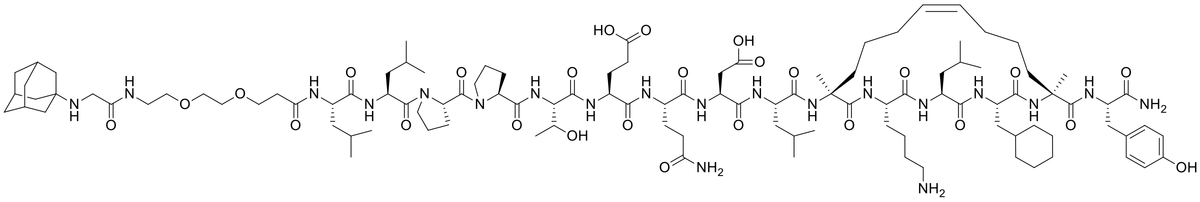

Calculated mass: 2181.31

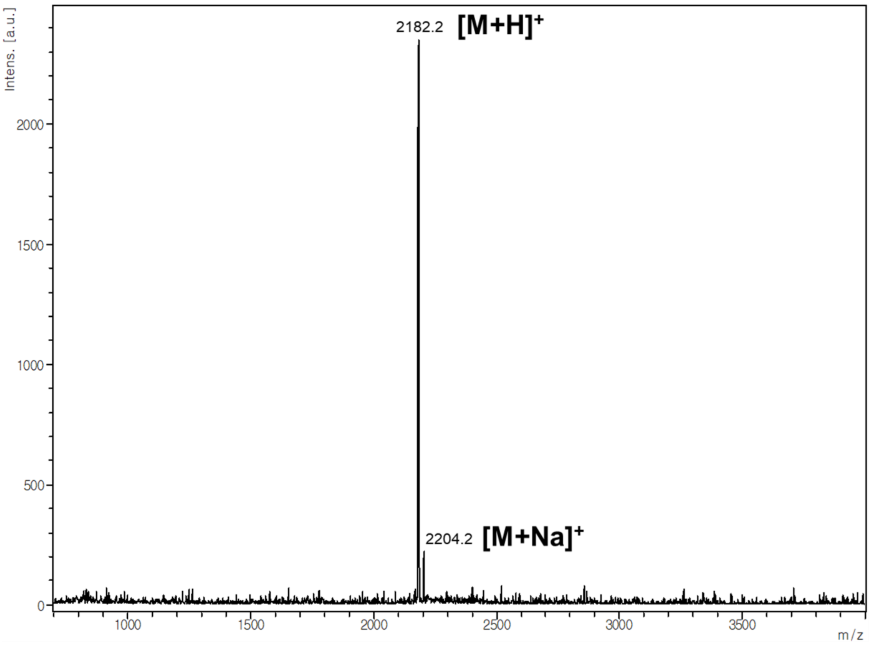

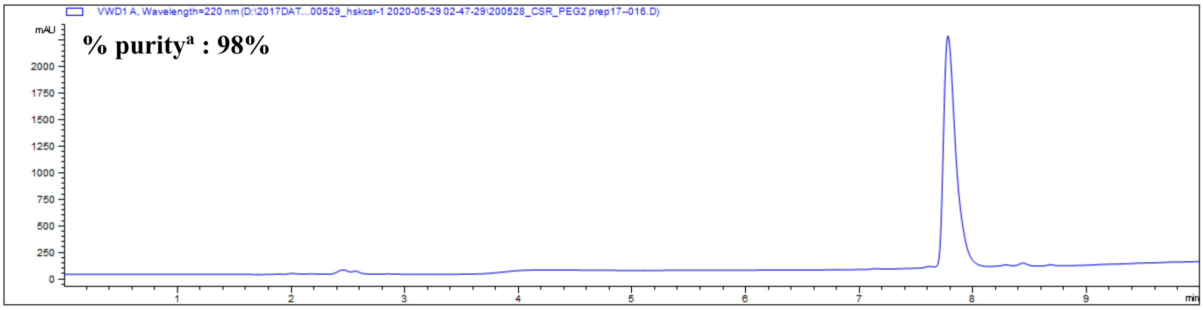

[a] Determined by analytical reversed-phase HPLC of a product

Figure S1. (Cont'd)

YL2-HyT6

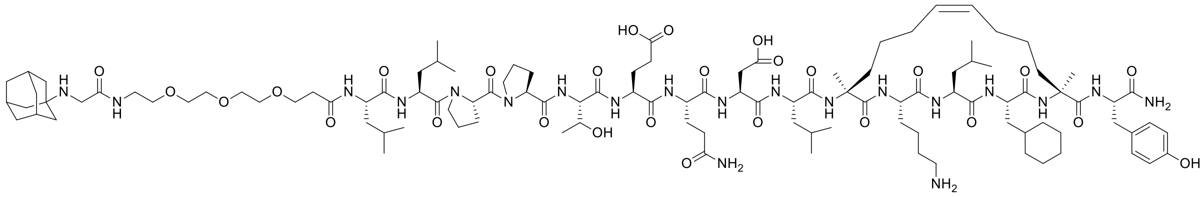

Calculated mass: 2225.33

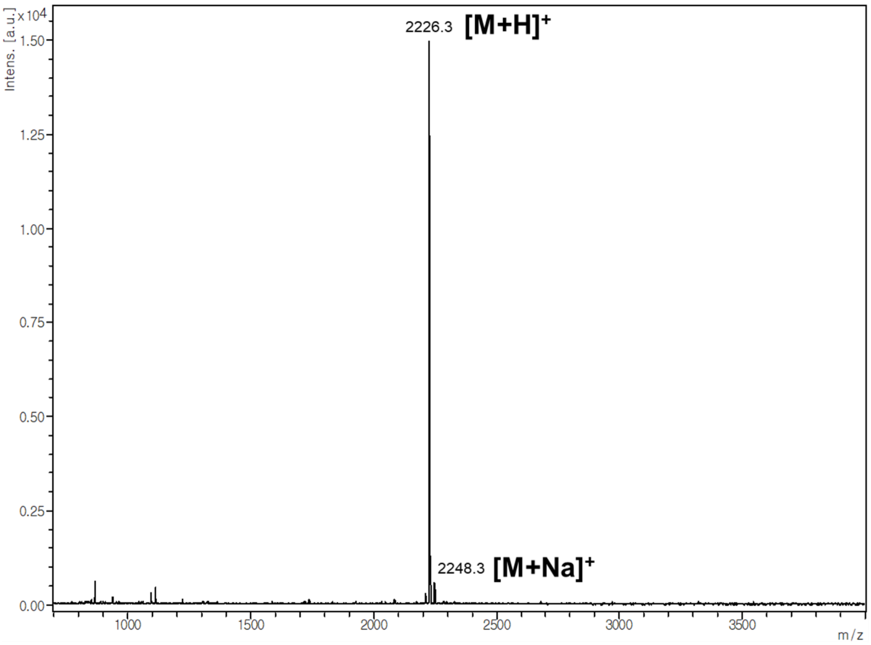

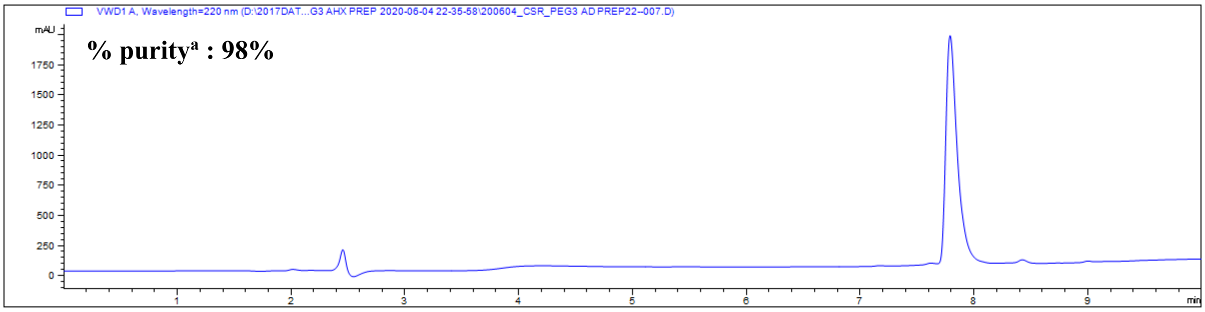

[a] Determined by analytical reversed-phase HPLC of a product

Figure S2. Western blot analysis of SRC-1 levels in MDA-MB-231 cells after treatment of YL2-HyT1 – 6 for 12 h. The SRC-1 levels (%) were normalized to GAPDH and DMSO controls.

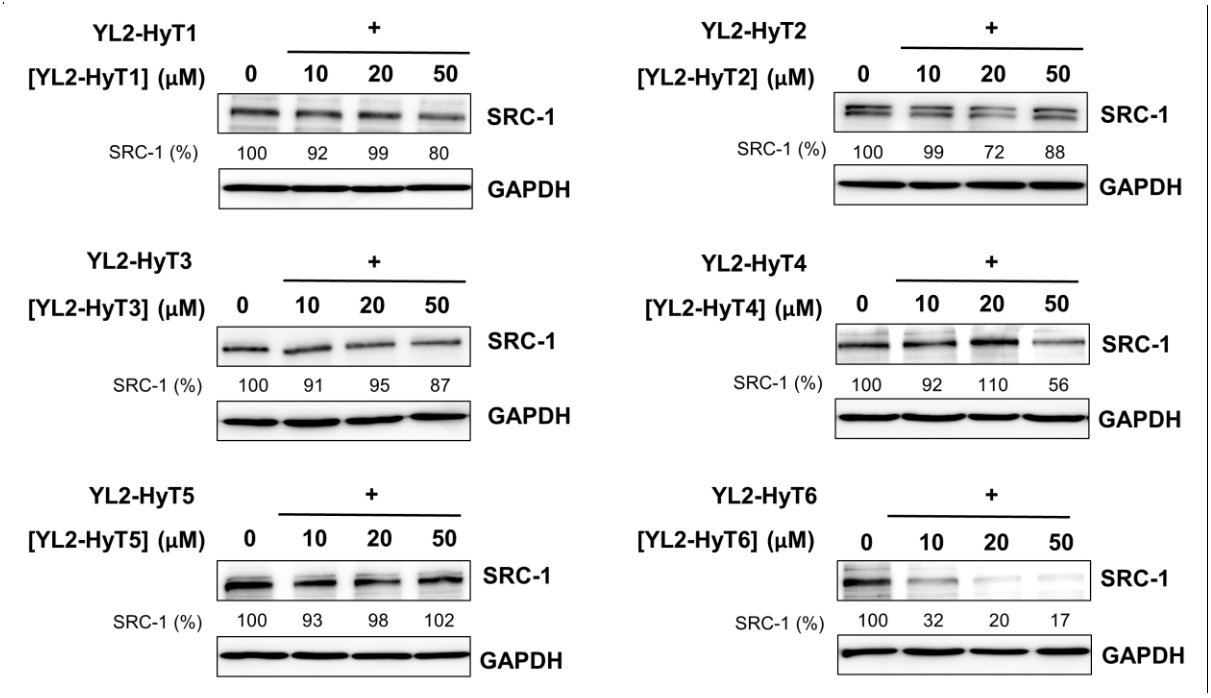

Figure S3. Western blot analysis of SRC-1 levels in MDA-MB-231 cells after treatment of YL2-HyT1 – 6 for 24 h. The SRC-1 levels (%) were normalized to GAPDH and DMSO controls.

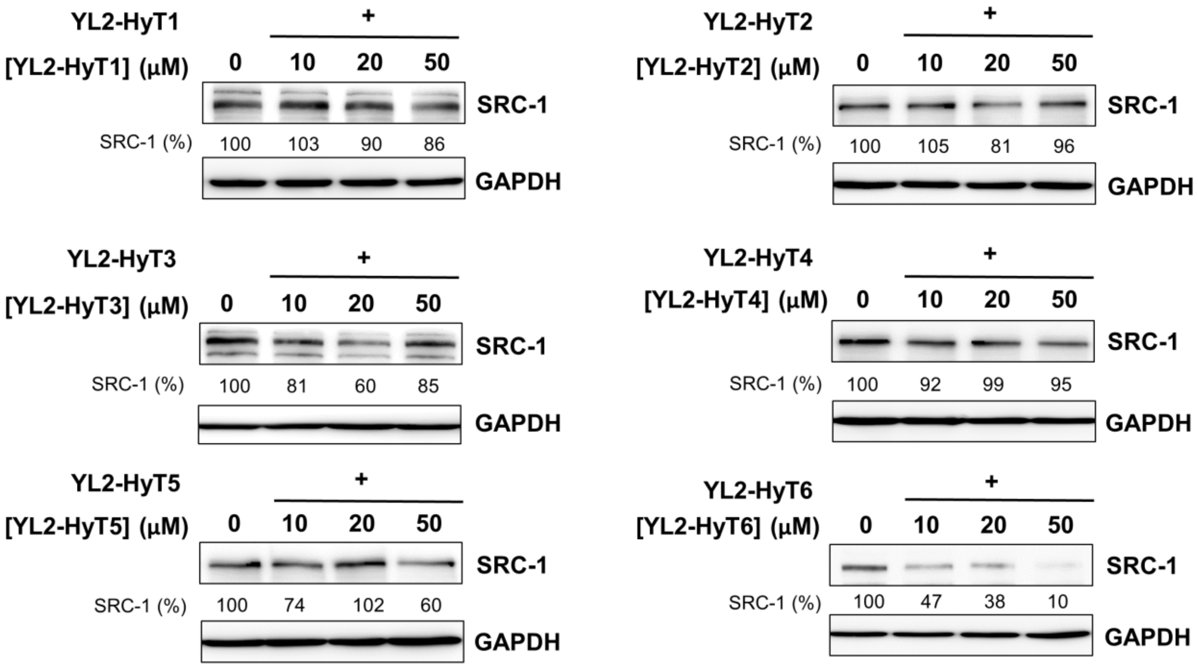

Figure S4. Quantitation of cellular levels of SRC-1 after incubation of MDA-MB-231 cells with indicated concentration of YL2-HyT6 for 18 h. The SRC-1 levels (%) were normalized to GAPDH and DMSO controls. Error bars in data represent standard deviation from three independent experiments.

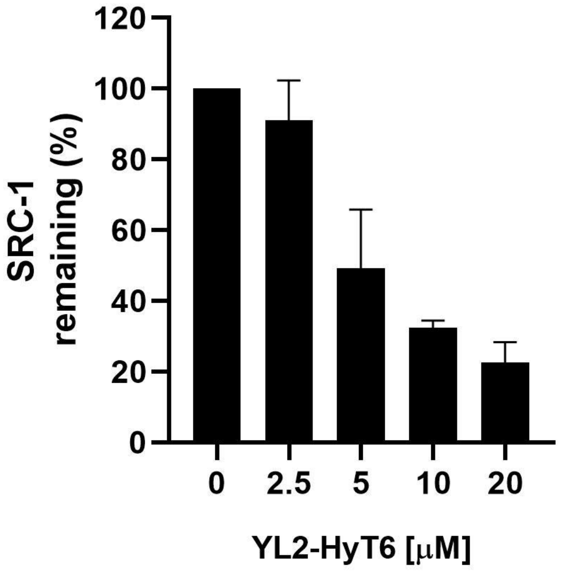

Scheme S2. Synthesis of TAMRA-labeled YL2-HyT6 (TAMRA-YL2-HyT6).

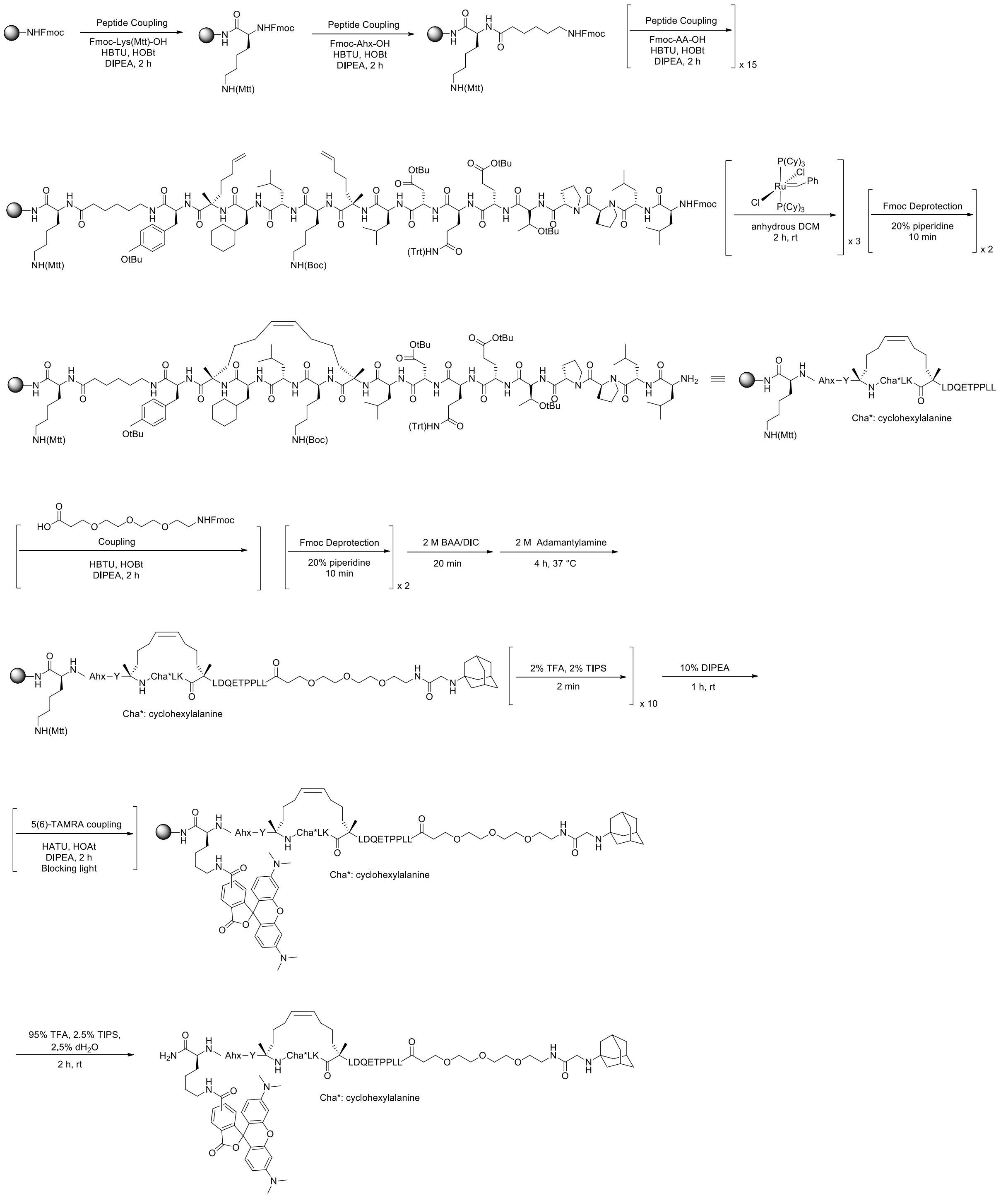

Scheme S3. Synthesis of TAMRA-labeled ND1-YL2 (TAMRA-ND1-YL2).

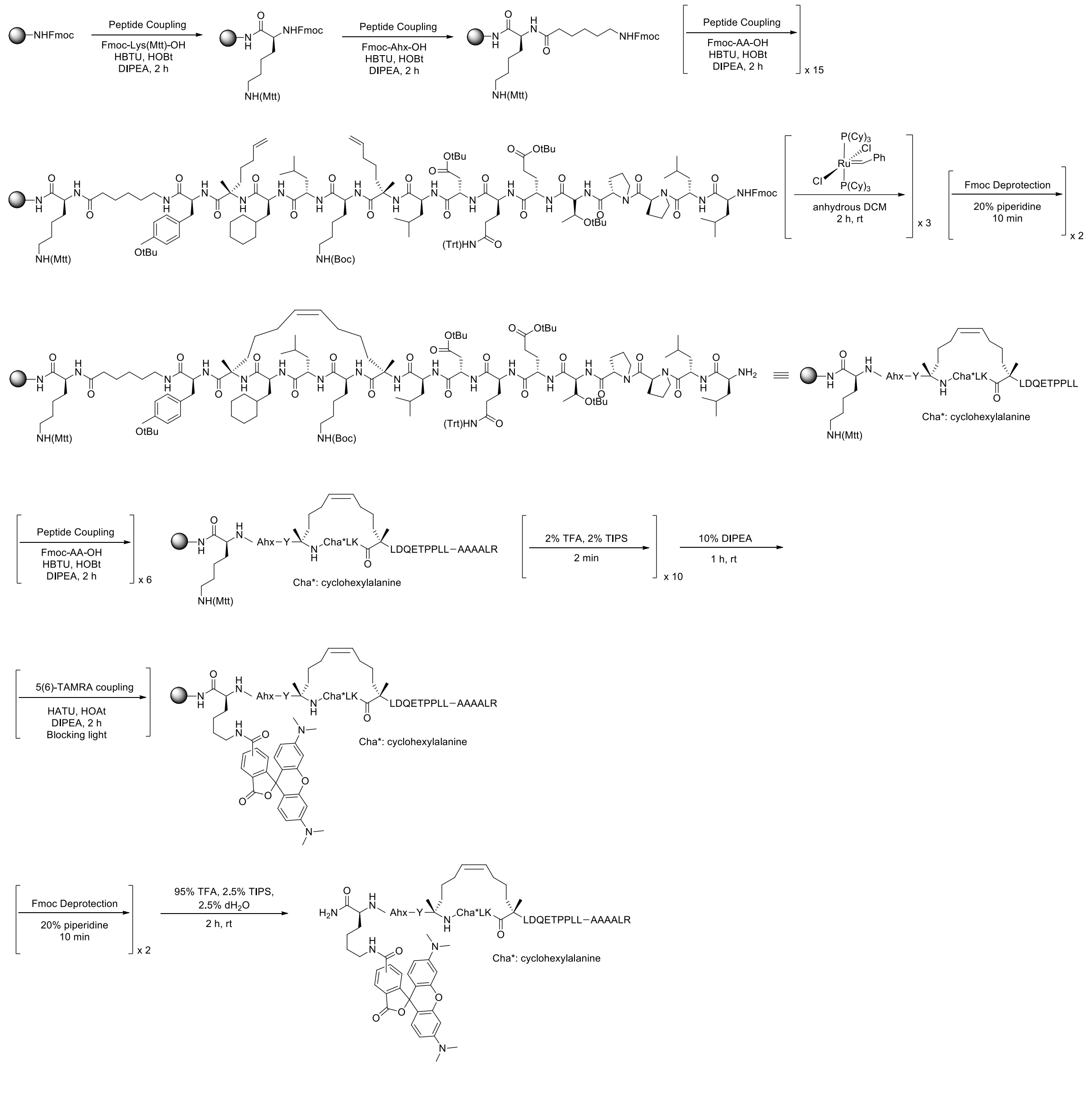

Figure S5. MALDI-TOF MS and LC data for purified TAMRA-YL2-HyT6 and TAMRA-ND1-YL2.

TAMRA-YL2-HyT6

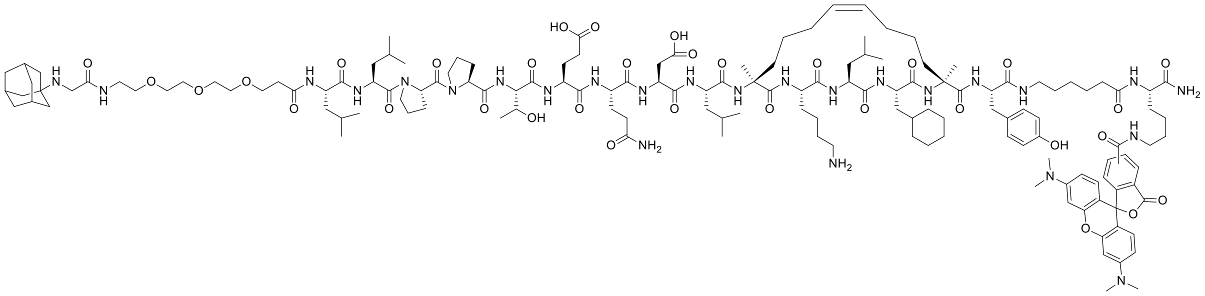

Calculated mass: 2878.65

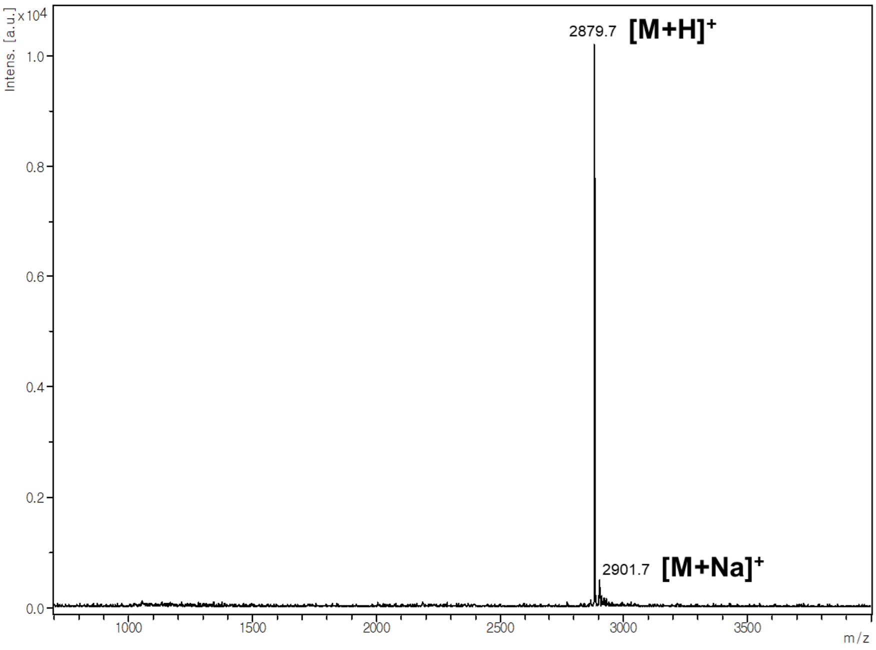

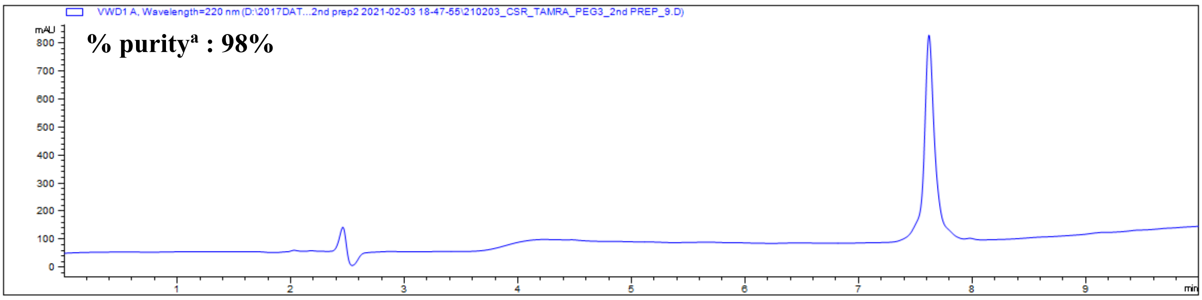

[a] Determined by analytical reversed-phase HPLC of a product

Figure S5. (Cont'd)

TAMRA-ND1-YL2

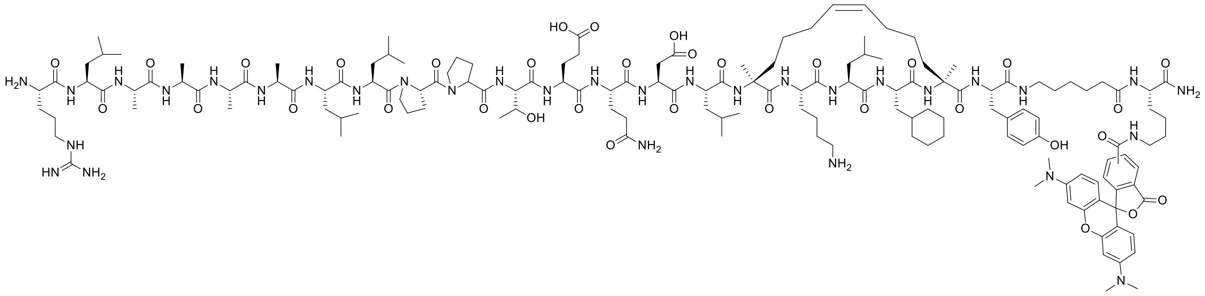

Calculated mass: 3037.74

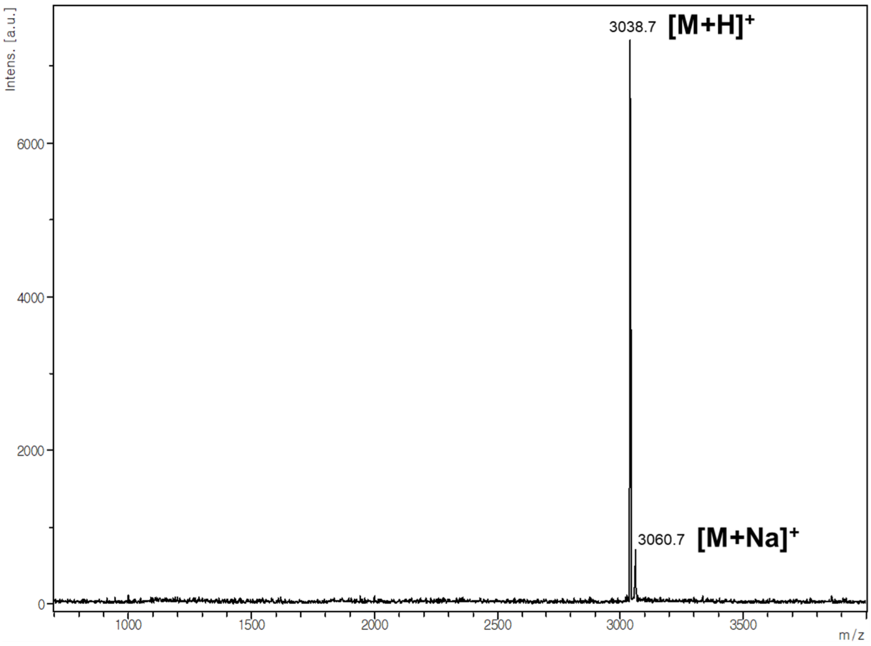

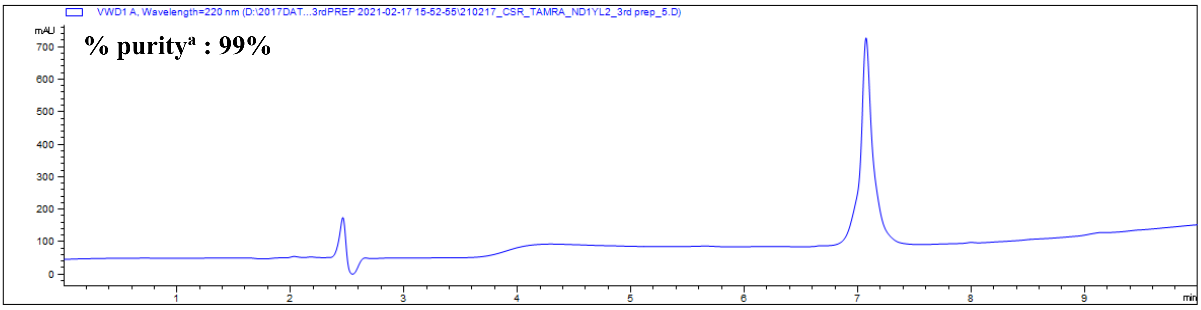

[a] Determined by analytical reversed-phase HPLC of a product

Figure S6. Representative image from wound healing assay showing changes in MDA-MB-231 cell migration after treatment with ND1-YL2 (20 μM). Images of wounds gap were taken at 0 h and 72 h.

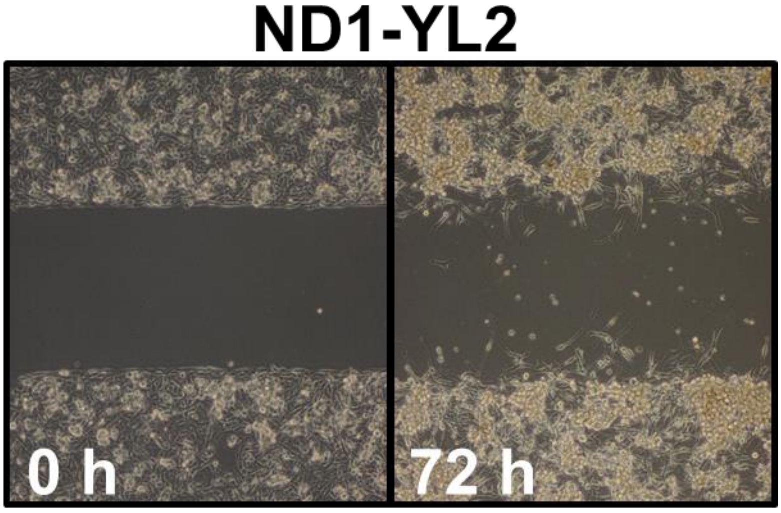

Figure S7. Representative image of transwell invasion assay of MDA-MB-231 cells after treatment with ND1-YL2 (20 μM) for 24 h.

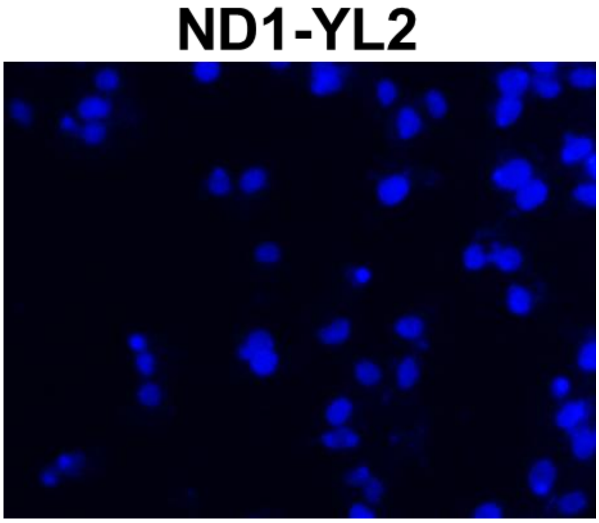

Supplement: Supplementary file 1 [file ijms-22-06407-s001.zip › ijms-1250613-supplementary.pdf]
